# Deregulation of the ubiquitin-proteasome system is the predominant molecular pathology in OPMD animal models and patients

**DOI:** 10.1186/2044-5040-1-15

**Published:** 2011-04-04

**Authors:** Seyed Yahya Anvar, Peter AC 't Hoen, Andrea Venema, Barbara van der Sluijs, Baziel van Engelen, Marc Snoeck, John Vissing, Capucine Trollet, George Dickson, Aymeric Chartier, Martine Simonelig, Gert-Jan B van Ommen, Silvere M van der Maarel, Vered Raz

**Affiliations:** 1 Center for Human and Clinical Genetics, Leiden University Medical Center, P.O. Box 9600, 2300 RC Leiden, the Netherlands; 2 Radboud University Nijmegen Medical Centre, Department of Neurology, Nijmegen, the Netherlands; 3 Department of Anaesthesia, Canisius-Wilhelmina Hospital, Nijmegen, the Netherlands; 4 Neuromuscular Research Unit and Department of Neurology, University of Copenhagen, Rigs Hospitalet, Denmark; 5 School of Biological Sciences, Royal Holloway - University of London, Surrey, TW20 0EX, UK; 6 INSERM U974, UMR 7215 CNRS, Institut de Myologie, UM 76 Université Pierre et Marie Curie, Paris, France; 7 Institut de Genetique Humaine, CNRS UPR1142, 141 rue de la Cardonille, 34396 Montpellier Cedex 5, France

## Abstract

Oculopharyngeal muscular dystrophy (OPMD) is a late-onset progressive muscle disorder caused by a poly-alanine expansion mutation in the Poly(A) Binding Protein Nuclear 1 (PABPN1). The molecular mechanisms that regulate disease onset and progression are largely unknown. In order to identify molecular pathways that are consistently associated with OPMD, we performed an integrated high-throughput transcriptome study in affected muscles of OPMD animal models and patients. The ubiquitin-proteasome system (UPS) was found to be the most consistently and significantly OPMD-deregulated pathway across species. We could correlate the association of the UPS OPMD-deregulated genes with stages of disease progression. The expression trend of a subset of these genes is age-associated and therefore, marks the late onset of the disease, and a second group with expression trends relating to disease-progression. We demonstrate a correlation between expression trends and entrapment into PABPN1 insoluble aggregates of OPMD-deregulated E3 ligases. We also show that manipulations of proteasome and immunoproteasome activity specifically affect the accumulation and aggregation of mutant PABPN1. We suggest that the natural decrease in proteasome expression and its activity during muscle aging contributes to the onset of the disease.

## Background

Oculopharyngeal muscular dystrophy (OPMD) is a late-onset progressive muscle disorder for which the underlying molecular mechanisms are largely unknown. This autosomal dominant muscular dystrophy has an estimated prevalence of 1 in 100,000 worldwide [1]. A higher prevalence has been reported in the Jewish Caucasian and French-Canadian populations (1 in 600 and 1 in 1,000, respectively) [2,3]. OPMD is caused by expansion of a homopolymeric alanine (Ala) stretch at the N-terminus of the Poly(A) Binding Protein Nuclear 1 (PABPN1) by two to seven additional Ala residues [4]. Although PABPN1 is ubiquitously expressed, the clinical and pathological features of OPMD are restricted to a subset of skeletal muscles, causing progressive *ptosis*, *dysphagia*, and limb muscle weakness. In affected muscles, the expanded PABPN1 (expPABPN1) accumulates in intranuclear inclusions (INI) [5]. Animal models for OPMD were generated in *Drosophila*, mice and *Caenorhabditis elegans* with a muscle-specific expression of expPABPN1 [6-8]. These models recapitulate INI formation and progressive muscle weakness in OPMD, and a correlation between INI formation and muscle weakness has been reported [6-8]. In these OPMD models protein disaggregation approaches attenuate muscle symptoms [8-10]. So far, however, the molecular mechanisms that are associated with OPMD onset and progression are not known. Previously, we performed transcriptome analysis on skeletal muscles from a mouse model of OPMD and found massive gene deregulation, which was reflected by a broad spectrum of altered cellular pathways [11]. We found an association of transcriptional changes with muscle atrophy [11]. Muscle atrophy was recently reported in homozygous OPMD patients [2]. However, the vast majority of OPMD patients are heterozygous and muscle atrophy is not a common pathological characteristic of the disease in its early stages. Importantly, a mouse model with low and constitutive expPABPN1 expression exhibits minor muscle defects without muscle atrophy [12]. Hino *et al. *[12] suggested that the extent of muscle symptoms caused by expPABPN1 depends on the expression level. Therefore, it is not known whether the massive transcriptional changes in affected muscles of the A17.1 OPMD model [11] are due to the high over-expression of expPABPN1 or if they are common with transcriptional changes in OPMD patients.

We have generated microarrays of OPMD carriers at pre-symptomatic and symptomatic stages. Since OPMD is categorized as a rare disorder in Western countries, limited patient material is an obstacle in reaching conclusive results. Therefore, we performed a cross-species transcriptome study by integrating transcriptome data from *Drosophila *and mouse models and heterozygous OPMD patients. We hypothesized that OPMD-associated molecular mechanisms would be consistently deregulated across species. As bioinformatics analyses of gene expression are biased by the computational approaches [13], here we integrated three computational methods to obtain a higher degree of confidence and reproducibility. The ubiquitin-proteasome system (UPS) was identified as the most significant and consistent OPMD-deregulated pathway across species.

## Results

Genome-wide expression profiles from the *Drosophila *and mouse OPMD models [6,11] were integrated with the expression profiles of heterozygous OPMD carriers (datasets are described in Additional file 1 Table S1 and Table S2). Genes that are differentially expressed between OPMD and controls (OPMD-deregulated) were identified using limma model in R [14]. To identify the most prominent and consistent feature across all species, comparative pathway analysis was performed using three computational methods (Additional file 1 Figure S1). In literature-aided analyses [15], the term 'ubiquitination' was found to be the most strongly associated biomedical concept with OPMD-deregulated genes (Table 1 and Additional file 1 Table S3). A regression-based analysis using global test (GT) [16], and an enrichment method using the Database for Annotation, Visualization, and Integrated Discovery (DAVID) [17,18] revealed highly significant deregulation of ubiquitin-proteasome system (UPS)-related GO (Gene Ontology) categories and KEGG (Kyoto Encyclopedia of Genes and Genomes) pathways across species (Table 1).

**Table 1:** Deregulation of ubiquitin-proteasome system (UPS) in OPMD in *Drosophila*, human and mouse.

	*Drosophila*a	Mouse	Human
**Literature Analysis**			
*Ubiquitination*	# 2	# 1	# 1
			
**GO Categories**			
*Ubiquitin-dependent Protein Catabolic Process*	2.81E-04	2.27E-07	1.22E-03
*Protein Ubiquitination*	7.57E-03	1.88E-05	9.24E-04
*Proteasomal Protein Catabolic Process*	6.51E-03	2.23E-07	1.86E-03
			
**KEGG Pathways**			
*Ubiquitin Mediated Proteolysis*	2.03E-03	8.25E-08	1.52E-03
*Proteasome*	2.15E-04	1.37E-07	9.27E-03

For *Drosophila *and mouse *P*-values are derived from the combined analysis of the three time points using global test, where age was included as a confounder in the model.

**Figure 1:**
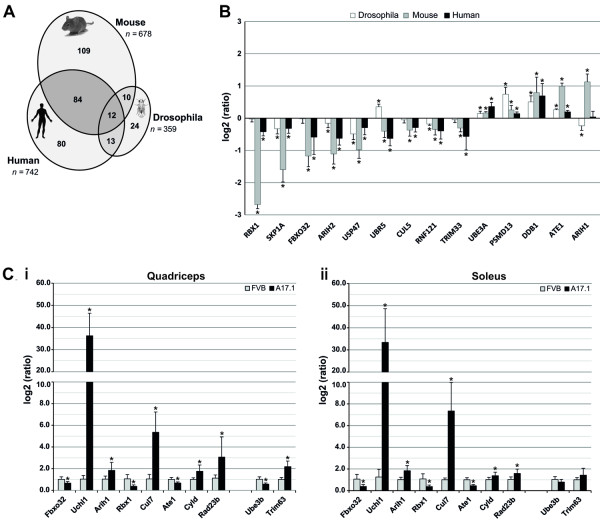
**Cross species deregulation of ubiquitin-proteasome in OPMD**. **A) **Venn-diagram displaying the overlap in OPMD-deregulated genes in UPS across species. In mice and *Drosophila*, OPMD-deregulated genes should be consistently deregulated in at least two time points. The total number of genes in UPS is indicated in italics. The list of OPMD-deregulated UPS genes is in Additional File 2. **B) **Transcriptional changes of selected genes in UPS in different organisms. Histograms display the log2(ratio) of the measured expression values in *Drosophila *(white bars), mice (gray bars), and humans (black bars). Significant changes with the adjusted *P *< 0.05 are indicated by *. **C) **RT Q-PCR validation of selected deregulated genes in UPS was carried out on quadriceps (**i**) and soleus (**ii**) muscles of six-week-old mice. Histograms show the measured expression values for A17.1 and FVB mice using Q-PCR. Significant changes of measured expression values of A17.1 mice as compared to FVB with the *P *< 0.05 are indicated by *.

To evaluate the level of concordance between the animal models and OPMD patients, gene overlap between the OPMD-deregulated UPS genes was determined. Homologous genes were annotated using the HomoloGene and Inparanoid databases (see Methods). In total, 16%, 32% and 25% of the genes annotated to the UPS were identified as OPMD-deregulated in *Drosophila*, mice and humans, respectively (Figure 1A). More than half of the OPMD-deregulated genes in *Drosophila *(59%) overlapped with their mouse or human homologous genes, and close to half (45% and 51%) overlapped between mouse and human genes, respectively (Figure 1A). The similarity of deregulation direction across species was demonstrated for 14 genes, for which probes were found in all organisms (Figure 1B). Similar transcriptional changes were found for 13 homologous genes in mouse and human datasets. Among those, eight genes showed similar changes in *Drosophila*. These results show the consistent UPS deregulation in OPMD.

To validate the microarray analyses, quantitative RT-PCR (Q-PCR) was performed on 19 OPMD-deregulated UPS genes from mice. Genes were selected based on *P*-value and >1.3-fold change criteria. For 17/19 genes (89%), Q-PCR results confirmed the results of the microarray analyses (Additional file 1 Figure S2). This demonstrates the reproducibility and validity of the microarray statistical analyses.

In the A17.1 mouse model, muscle atrophy is more prominent in fast glycolytic fibers (quadriceps) as compared with slow oxidative fibers (soleus) [11]. Since muscle atrophy is regulated by the UPS [19-21], we analyzed the muscle-type specific expression of 10 OPMD-deregulated UPS genes in order to identify a correlation with muscle atrophy. Q-PCR was performed on RNA isolated from quadriceps and soleus of six-week-old A17.1 and control (Friend Virus B inbred (FVB)) mice. The majority (8 out of 10) of genes showed no fiber-type specificity (Figure 1C). Only the deregulation of *Trim63 *[11] and *Ube3b *were specific to fast glycolytic fibers (Figure 1C). This suggests that the majority of OPMD-deregulated UPS genes are not associated with muscle atrophy in the A17.1 mouse.

**Figure 2:**
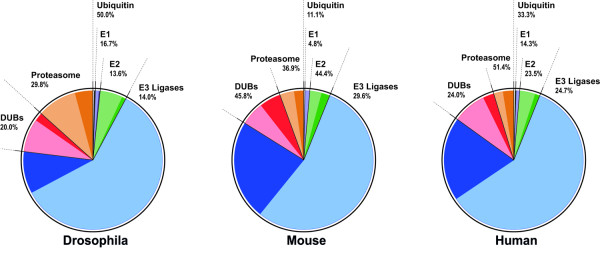
**OPMD deregulated genes in the UPS**. Pie charts show the relative distribution of the UPS units (light colors) and OPMD-deregulated genes (dark colors) per organism. Numbers indicate the percentage of OPMD-deregulation.

The UPS involves an enzymatic cascade of ubiquitination and degradation steps. The ubiquitination steps start with ubiquitin activation, which requires the ubiquitin-activating enzyme (E1) and ubiquitin (Ub). This process results in the binding of Ub to the E2-conjugating enzyme. In a subsequent step the target protein is ubiquitinated with Ub-E2 and E3-ligase complexes, which ensures target specificity. Poly-ubiquitinated proteins are subjected to degradation. This step is employed by the deubiquitinating enzymes (DUBs) and the proteasome [22,23]. Deregulation of genes involved in the ubiquitin activation step was not found to be consistent between OPMD and the models (Figure 2 andTable 2). Ubiquitin up-regulation was previously reported in a non-muscle cell model for OPMD [24]. Our study identified only one ubiquitin-encoding gene to be up regulated in mouse and human genomes, but these deregulated genes were not consistent across species. The E2-conjugating enzymes were significantly deregulated in *Drosophila *and mouse genomes, whereas in humans, the *P*-value for these enzymes was not significant. This suggests a weak association of E2 deregulation with OPMD (Figure 2 andTable 2). In contrast, consistent deregulation was found for E3-ligases, DUBs, and proteasome (Figure 2 andTable 2). The significance of this strong association was further evaluated by gene-overlap of homologous genes in humans and mice (Table 2). The gene overlap between mice and humans was found to be significant for all these three UPS components (*P*-values are 6.64E-08 for E3-ligases, 1.37E-02 for DUBs and 1.70E-02 for the proteasome). Overall, this analysis demonstrates consistent deregulation of E3-ligases, DUBs and proteasome across species.

**Table 2:** The distribution of OPMD-deregulated genes in UPS functional units and protein degradation categories.

	*Drosophila*			Mouse			Human			*Overlap mouse vs. human*	
	**# Total Genes**	**% D.E. Genes**	***P*Value (FDR)**	**# Total Genes**	**% D.E. Genes**	***P*-Value (FDR)**	**# Total Genes**	**% D.E. Genes**	***P*-Value (FDR)**	**# D.E. Genes**	**% D.E. Genes**
**Ubiquitin**	2	50.00	4.18E-05	3	11.11	1.28E-01	3	33.33	1.19E-01	0	00.00
**E1 Ubiquitin Activation**	4	16.67	1.24E-01	7	04.76	7.29E-02	7	14.29	7.94E-02	0	00.00
**E2 Ubiquitin Conjugation**	22	13.64	9.19E-06	33	44.42	1.51E-08	34	23.53	7.31E-02	3	37.50
**E3 Ubiquitin Ligase**	249	13.99	1.92E-04	526	29.58	1.64E-08	586	24.74	4.35E-03	69	47.59
**Deubiquitination (DUB)**	35	20.00	1.63E-05	72	45.83	1.48E-08	75	24.00	3.15E-02	13	72.22
**¥ **Proteasome	47	29.79	2.15E-04	37	36.94	1.37E-07	37	51.35	9.27E-03	11	57.90
**¥ **Autophagy	16	25.00	1.07E-03	39	30.77	8.13E-08	32	18.75	1.37E-02	1	16.67
**¥**Lysosome	60	5.00	1.64E-02	75	25.33	6.06E-03	73	24.68	1.54E-02	6	33.33

The number of annotated genes per unit, the percentage of OPMD-deregulated (D.E.) genes and *P-*values are indicated per organism. For *Drosophila *and mouse statistics is generated in combined datasets from three time points. The overlap in OPMD-deregulated genes between humans and mice and the percentage of deregulated genes in human D.E. genes are indicated. Protein degradation machineries are depicted by ¥.

**Figure 3:**
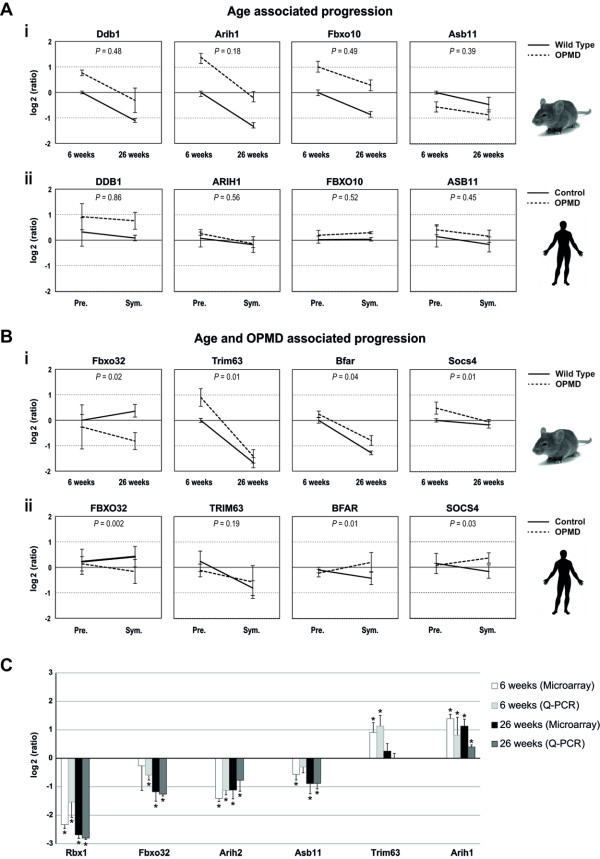
**Progressive changes in UPS gene expression**. Progression trends for selected genes in mice (**i**) and humans (**ii**). Expression values were normalized to six-week-old WT in mice, and to young healthy controls (19 years old on average) in humans. *P*-values demonstrate the significance of differences in expression trends between controls and OPMD samples. **A) **The age-associated progression trend is indicated by *P*-value >0.05. **B) **The genotype-specific progression trend is indicated by *P*-values <0.05. SD represents variations in mice (6 weeks N = 5 and 26 weeks N = 6) and in humans (expPABPN1 carriers N = 4 and controls N = 5). **C) **RT Q-PCR validation of selected deregulated genes in UPS was carried out on skeletal muscles of 6-week-old and 26-week-old mice. Histograms show the log2(ratio) of the measured expression values using microarray and Q-PCR. Significant changes with the *P *<0.05 are indicated by *.

OPMD is characterized by a late onset and a slow progression of muscle weaknesses [4,25]. Progressive muscle weakness has also been reported in the mouse model [7]. In 6-week-old mice symptoms were not detected, while muscle weakness was present in 18-week-old mice and was more pronounced by 26 weeks [7]. If changes in expression levels are associated with disease onset and progression, a correlation between age and expression levels should be expected. A linear regression model was applied to the mouse UPS genes at three time points in order to identify genes that their expression trends are progressively changed. 80% of the OPMD-deregulated UPS genes show a progressive trend, which is age-associated (N = 171/217, Additional file 1 Figure S3A, examples for progressive expression trends are shown in Figure 3Ai). To identify genes with expression trends that are specific to the disease a regression model that combines age and disease features was applied. In 30% of the age-associated OPMD-deregulated UPS genes (N = 50, Figure S3B) the progression trends significantly (*P*-value <0.05) differed between A17.1 and the wild-type (WT) controls (examples for progressive expression trends are shown in Figure 3Bi). The genes with disease-specific progression can be used to mark disease progression and could contribute to disease onset and progression. The group of genes whose expression changes with age independent from the disease, however, may contribute to the late onset of the disease.

The vast majority of OPMD-deregulated UPS genes, which exhibit progressive expression profiles encode for E3-ligases (Additional file 1 Figure S3). Expression trends for selected E3-ligases are presented in Figure 3. Confirmation of the analysis in mice was carried out on the human homologues (Figure 3). The age-associated expression trends were similar between A17.1 and WT in mice and between controls and expPABPN1 carriers at pre-symptomatic and symptomatic stages in humans (Figure 3A). The progression trends did not significantly differ between genotypes (*P*-value >0.05). In contrast, for those genes with expression trends associated with age and disease the expression trends of controls significantly differed from those of OPMD subjects (Figure 3B, P-value <0.05). Validation of progression analysis was performed by Q-PCR analysis of RNA from 6- and 26-week-old mice (Figure 3C). The Q-PCR results demonstrate the reproducibility and validity of the microarray progression analysis.

In the progression analysis some differences between humans and mice were noted. The progression of *Trim63 *is mouse-specific, whereas the expression of the human *TRIM63 *is not age-associated or OPMD-deregulated (Figure 3B). *Asb11 *is down regulated in mice while it is up regulated in humans (Figure 3A). The expression trend of *Socs4 *in mice is negative while in humans it is positive (Figure 3B). These discrepancies could reflect differences between the two organisms or between the heterozygous and the high over-expression situation.

**Figure 4:**
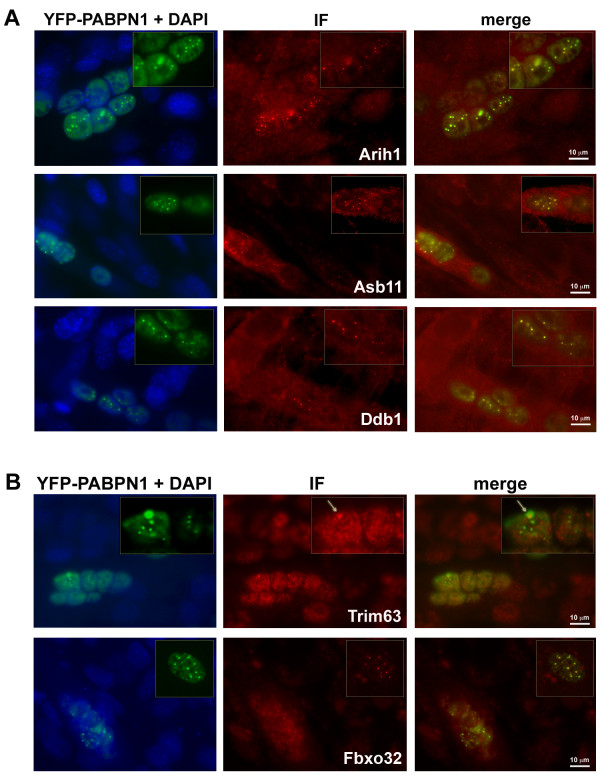
**Co-localization of selected E3 ligases with INI in C2C12 myotubes expressing YFP-Ala16PABPN1**. Immunostaining of E3-ligases was visualized with Alexa-594 secondary antibodies. Co-localization with expPABPN1 in myotubes is demonstrated in the merge image. A 2.5X magnification of nuclei containing expPABPN1 aggregates is highlighted in a box. **A) **Arih1, Asb11 and Ddb1 E3 ligases show consistent co-localization with aggregated YFP-Ala16-PABPN1. **B) **Trim63 and Fbxo32 E3 ligases show progressively more co-localization with YFP-Ala16-PABPN1 as INI size increases. Scale bar is 10 μm.

Expression of expPABPN1 leads to INI formation in affected muscles [7,11]. Previous studies have demonstrated that ubiquitin and proteasome proteins co-localize with INI in affected muscles [26] and in non-muscle cells [24,27]. Since INI formation is a hallmark of OPMD, we studied whether the expression profiles of OPMD-deregulated E3-ligases correlate with their entrapment with expPABPN1 in INI. Co-localization was analyzed with an immunofluorescence procedure in C2C12 myotubes expressing expPABPN1 fused to yellow fluorescent protein (YFP). From the E3-ligases encoding genes that showed an association with disease onset or progression (Figure 3), five were selected for co-localization studies using specific antibodies recognizing single proteins at the appropriate molecular weights. All five proteins showed nuclear localization in myotubes and co-localized with expPABPN1 in INI (Figure 4). Arih1, Asb11 and Ddb1 co-localized with all sizes of INI structures (Figure 4A) while the co-localization of Trim63 and Fbxo32 proteins were only evident for larger INI structures (Figure 4B, highlighted in boxes). This suggests a correlation between changes in expression trends and temporal entrapment in INI.

**Figure 5:**
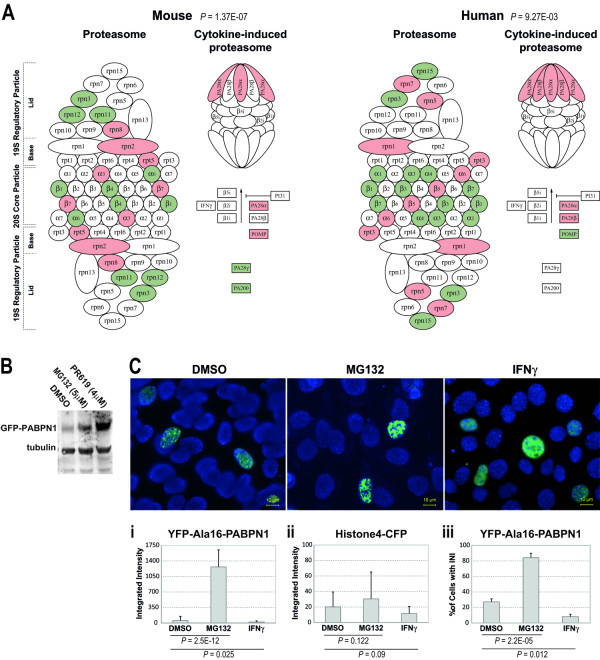
**The effect of altered proteasome activity on expPABPN1 accumulation and aggregation**. **A) **Substantial deregulation of proteasome and immunoproteasome encoding genes in mice and humans. Down-regulation (green) is more pronounced in the core subunit of the proteasome. Immunoproteasome shows consistent up-regulation (red) in both organisms. **B) **Western blot analysis of YFP-Ala16-PABPN1 transfected C2C12 cells that were treated with 5 μM MG132 or 5 nM PR619. Control cells were treated with DMSO. **C) **Images show YFP-Ala16-PABPN1 localization in C2C12 after mock-treatment (DMSO), 5 μM MG132 or 5 U/ml IFNγ. Scale bar equals 10 μm. Histograms show the integrated intensity of YFP-Ala16-PABPN1 (**i**) or Histone4-CFP (control) (**ii**), and the percentage of cells with INI in YFP-Ala16-PABPN1 expressing cells (**iii**). Averages represent 509, 773 and 476 cells for DMSO, MG132 and IFNγ, respectively. Significant difference between treatments is reflected by *P*-values.

The proteasome is composed of core and regulatory subunits. Genes encoding for the proteasome core subunit were prominently down regulated in mice and humans (66% and 75%, respectively), while no preference in deregulation direction was found for the regulatory subunit (Figure 5A andAdditional file 1 Table S5). Down-regulation of the proteasome could affect protein degradation and, hence, protein accumulation. In C2C12 myoblasts that were treated with low concentrations (5 μM) of the proteasome inhibitor MG132, the accumulation of expPABPN1 was significantly higher as compared with mock-treated cells (Figure 5B). Similarly, treatment with the DUB inhibitor, PR619, also caused expPABPN1 accumulation (Figure 5B). High nuclear accumulation of expPABPN1, which accompanies INI formation, was consistently measured in MG132-treated cells using a cell-based intensity fluorescence quantification assay (Figure 5C). Thus, reduced proteasome and DUB activities in muscle cells promoted expPABPN1 accumulation and INI formation in muscle cells. However, expPABPN1 accumulation stimulated by proteasome inhibition is not specific to muscle cells [24].

In addition to the proteasome, the lysosome and the autophagy machineries can also facilitate protein catabolism. To evaluate whether one of these machineries could also regulate expPABPN1 protein accumulation, the significance of deregulation in OPMD was analyzed. Overall, deregulation of lysosome and autophagy were not consistent across species. The lysosome KEGG pathway was evaluated as significantly deregulated in OPMD across species by GT but not by DAVID analysis (Table 2). However, in the literature-aided analysis, only a low level of association was found between OPMD-deregulated genes and lysosome in *Drosophila *and humans (ranked at positions 196 and 789, respectively), while no association was found in mice. Similarly, the autophagy KEGG pathway was significant across species based on GT but not on DAVID analysis (Table 2). In the literature-aided analysis, autophagy was ranked 12 in mice, but a lower priority (ranked 136 and 154) was found in *Drosophila *and humans, respectively. Furthermore, the OPMD-deregulated gene overlap between mice and humans was not significant for either lysosome or autophagy pathways (*P*-values: 5.37E-01 and 3.70E-01, respectively). This is in sharp contrast to the consistent proteasome deregulation found across species. This indicates that, from the protein degradation pathways, only proteasome deregulation is consistently associated with OPMD across species. From this analysis we cannot exclude lysosome or autophagy deregulation in OPMD, but the lack of consistency across species and in three bioinformatics analyses suggests a smaller contribution as compared with the proteasome.

In contrast to the down-regulation of genes in the core-subunit, the expression of genes encoding for the immunoproteasome subunit (the cytokine-induced proteasome) was consistently elevated in OPMD (Figure 5A). The immunoproteasome was initially identified in cells of the immune system after cytokine induction, which is involved in MHC-class-I antigen presentation [28]. However, the accumulation of cytokine-induced proteasome proteins was also found in aging skeletal muscle cells [29]. Treatment of C2C12 myoblasts with IFNγ, an inducer of immunoproteasome activity [30], led to a significant reduction in nuclear expPABPN1 accumulation (Figure 5C, Ci) and INI formation (Figure 5Ciii). In contrast to expPABPN1, accumulation of Histone4, which is also a nuclear protein, was not significantly affected by manipulation of proteasome activity (Figure 5Cii). This suggests that the accumulation of expPABPN1, but not of Histone4, is receptive to the level of proteasome and immunoproteasome activity. Together, our results demonstrate that the UPS degradation machinery regulates expPABPN1 accumulation.

## Discussion

UPS is a cellular regulator of homeostasis and is involved in a wide spectrum of human diseases including cancer, neurodegenerative disorders and diabetes [31-35]. Deregulation of UPS has been reported for myotonic dystrophy type 1 [36] and muscle atrophy in mice [19-21]. In addition, altered UPS activity has been associated with muscle aging [33,37]. Together these studies suggest that muscle cell function is tightly regulated by the UPS. In this study, we identified the UPS as the most consistently and significantly deregulated cellular machinery in OPMD animal models and patients. Transcriptome studies in non-muscle cells expressing expPABPN1 did not reveal substantial and predominant deregulation of UPS genes [38]. This indicates that the effect of expPABPN1 on UPS deregulation is specific to muscle cells. Since PABPN1 is ubiquitously expressed in every cell but the phenotype is limited to muscle cells this suggests that UPS deregulation confers the muscle-specific pathogenesis of OPMD.

**Figure 6:**
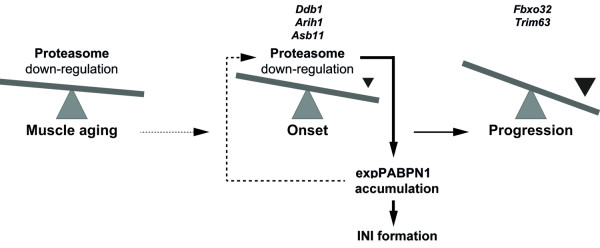
**A model for the involvement of UPS in OPMD disease pathology**. In muscle, age-associated proteasome down-regulation affects expPABPN1 protein accumulation. Elevated expPABPN1 accumulation affects proteasome deregulation during disease onset. Expression profiles of E3-ligases can be sued to separate disease onset from progression.

From six UPS components, only E3-ligases, DUBs and the proteasome were found to be consistently and prominently deregulated in OPMD across species. Relevant to OPMD proteasome activity is reduced during muscle aging [29,33,37], and is associated with transcriptional deregulation of proteasomal genes [37]. In the analysis of expression trends the expression of 89% of the OPMD-deregulated proteasome genes were found to be age-associated. This suggests that the natural decrease in proteasome expression during muscle aging can contribute to the late onset of the disease. Our analysis revealed that the core subunit of the proteasome is the only UPS subunit that was consistently down-regulated which can cause reduced activity of the proteasome machinery. In a recent study, we found that expression of expPABPN1 in myotubes leads to down-regulation of proteasome-encoding genes, and causing the accumulation of expPABPN1 protein (unpublished data). However, proteasome regulation of expPABPN1 accumulation and INI formation is not specific to muscle cells [24]. Since in patients INI are formed only in muscle cells, this suggests that proteasome down-regulation during muscle aging triggers expPABPN1 accumulation. In turn, accumulation of expPABPN1 leads to extensive proteasome down-regulation in OPMD (Figure 6). This feed-forward model could justify the muscle-specific INI formation and the late onset in OPMD.

Hypothesizing that changes in expression levels could reflect pathological changes in disease status we have studied the correlation between transcriptional changes of OPMD-deregulated UPS genes and age. Noticeably, the expression of the vast majority of the OPMD-deregulated UPS genes is progressed during normal muscle aging. This suggests that transcriptional changes of these genes are associated with disease onset. The expression trends of a subset of these genes showed disease-specific progression. Among those, *Trim63 *and *Fbxo32 *exhibited disease progression in mice. Both genes are known for regulating muscle atrophy in mice [19-21]. In the OPMD mouse model, muscle atrophy in exhibited only in fast muscle glycolytic fibres and *Trim63 *expression correlates with muscle atrophy in A17.1 [11]. However, the majority of the OPMD-deregulated UPS genes did not show fibre-type specific expression. This could suggest that UPS deregulation in OPMD has a broader pathological effect than muscle atrophy. Indeed, in affected muscles of OPMD patients, atrophy may be evident only at a later stage of disease progression; although a high degree of consistency between expression trends in mice and humans was found for the majority of the genes analyzed in this study. *Trim63 *deregulation and progression is probably mouse-specific, as OPMD-deregulation or progression was not found in humans. *Fbxo32*, however, was consistently deregulated in both organisms and, therefore, can be a candidate for regulating disease progression and muscle atrophy in humans. After mining the NCBI dataset for tissue-specific expression (Unigene Hs.352183, Build No. 228 released 2010), *Asb11 *was noted for its specific expression in skeletal muscles. Since *Asb11 *is consistently OPMD-deregulated in humans and mice, and its expression trend is associated with disease onset it could represent a relevant candidate for functional genomic studies. This shows that cross-species transcriptome and progression analyses can be used to identify target molecules for future studies.

OPMD is characterized by INI formation. The role of INIs in disease pathogenesis is unknown. Previous studies have shown that many genes whose expression is deregulated by expPABPN1 are found to be co-localized in INI [38]. Components of the proteasome, which is OPMD-deregulated, also co-localize in INI [24,27]. We also found that many of the OPMD deregulated E3-ligases are entrapped in INI. Moreover, we demonstrate a correlation between temporal changes in expression levels and sequential entrapment in INI. Together these studies suggest that entrapment in INI could lead to transcriptional deregulation. It is possible that protein entrapment in INI affects gene expression through a compensatory mechanism resulting in altered transcriptional profiles.

## Conclusions

In this study, we combined expression datasets from three organisms and disease models with different bioinformatics analyses in a single study. This allowed us to identify with high confidence the UPS as the most predominantly deregulated cellular pathway in OPMD. This approach differs from most microarray studies where results are derived from a single computational analysis performed on a single organism. We show that with this combined bioinformatics approach the list of deregulated pathways can be prioritized with high confidence. This approach can facilitate studies with complex biological situations and massive gene deregulation, such as late onset disorders and rare-diseases.

The most significant and novel finding in this study is the substantial and cross-species consistent deregulation of the UPS in OPMD. We propose that protein entrapment in PABPN1 aggregates is associated with a substantial transcriptional deregulation of the UPS that, in turn, leads to disruption of homeostasis in skeletal muscles. By taking advantage of the detailed analysis of gene expression trends and muscle-expression, we predict that candidate genes can be selected for functional genomic studies which ultimately lead to the identification of OPMD pathogenesis.

## Methods

### Generation of microarray datasets

*Drosophila *and mouse microarray datasets have previously been published [10,11]. Human quadricep muscle samples were collected with the needle or by an open surgical procedure from OPMD patients and family members as well as from anonymous age-matching healthy individuals that gave informed consent. The presence of expansion mutation in PABPN1 in OPMD patients and pre-symptomatic individuals was determined with sequencing. Bergstrom needle biopsies from the (pre)symptomatic patients were approved by the ethical committee. Total RNA was extracted from skeletal muscles using RNAease Bee (Amsbio, Abingdon, United Kingdom) according to the manufacturer's instructions. RNA integration number (RIN) was determined with RNA 6000 Nano (Agilent Technologies Amstelveen, The Netherland). RNA with RIN >7 were used for subsequent steps. RNA labeling was performed with the Illumina^® ^TotalPrep RNA Amplification kit (Ambion, Austin, TX, United States) according to the manufacturer's protocol, and subsequently was hybridized to Illumina Human v3 Bead arrays. The generated microarray datasets are deposited and publicly available at GEO repository. GEO accession numbers for mouse and human microarray datasets are GSE26604 and GSE26605, respectively.

### Data processing and statistical analysis

Microarray measurements were normalized using the quantile method [39]. Each organism and time point was normalized separately. The quality of the data was assessed by principal component analysis.

For *Drosophila *and mice, genes differentially expressed between OPMD and control subjects were identified at each time point by applying a hierarchical linear model using the limma package in R [14]. Human subjects were grouped into healthy, pre-symptomatic and symptomatic subjects. *P*-value cut-offs of 0.05 after multiple-testing correction using the method of Benjamini and Hochberg (False Discovery Rate (FDR) were applied to the *Drosophila *and mouse samples and, due to higher inter-individual variation, a nominal *P*-value cut-off of 0.05 was used for human samples) were used. This resulted in lists of OPMD-deregulated genes for each time point and organism. Probe annotation was done using the indac (*Drosophila*), illuminaMousev1BeadID (mouse), and illuminaHumanv3BeadID (human) R packages. The OPMD significantly deregulated genes in the UPS from human and mouse datasets are listed in Additional File 2.

### Pathway analyses

GT [16] was used to identify significant associations between GO categories or KEGG pathways and OPMD, while including age as a confounder (*Drosophila *and mice only). Gene sets with multiple testing adjusted (Holm's method) *P-value *<0.05 were selected as significant. DAVID, a functional annotation clustering tool [17,18], was applied on a list of OPMD-deregulated genes and pathway redundancy was removed by clustering similar GO categories and pathways. In addition, biomedical concepts that are associated with OPMD-deregulated genes were identified using a literature-aided mapping tool, Anni 2.0 [15]. The procedure was performed for each organism separately. Cross-species analyses were carried out on a group of homologous genes. *Drosophila *homologues of mouse and human genes were annotated using HomoloGene http://www.ncbi.nlm.nih.gov/homologene and Inparanoid http://inparanoid.sbc.su.se online databases. Integration of three time-points in *Drosophila *and mice (Additional file 1 Table S1) were used to identify OPMD-deregulated pathways across species (Figure 1). A recent annotation of E3 ligases [40] was used to identify OPMD-deregulated E3 ligases. The annotation for all other UPS components is extracted from KEGG. Since the annotation for genes encoding for lysosome is not available in R packages, we have extracted the annotation from KEGG website and integrated it into our pathway analyses.

### Progression studies

For testing the significance of the association of expression trends of OPMD-deregulated genes with age, using limma model in R [14], a linear regression model (*expression *~ α*OPMD *+ *βAGE *+ δ(*OPMD *× *AGE*) + *ε*) was applied on combined datasets from 6- and 26-week-old mice. Age-associated changes were identified as those with β significantly different from zero. OPMD- and age-associated changes were defined as those with δ significantly different from zero. To determine whether the expression profiles of individual genes significantly differ between controls and OPMD *P*-values are FDR-corrected with the cut-off threshold of 0.05.

### Quantitative RT-PCR analysis

Primers for Q-PCR validation were designed in the sequence surrounding the Illumina probe location using Primer 3 plus program. RT-QPCR was performed according to the procedure in Trollet *et al. *[11]. The list of primers is provided in Table 3.

**Table 3:** The list of primers used for quantitative RT_PCR analysis.

Genes	Probe	FW Primer Sequence	RV Primer Sequence
**Arih1**	6900025	GAGAAGGATGGCGGTTGTAA	ATCTCTTGCTGCCTTTGCAT
**Arih2**	2810025	AGCCTAACTCCCCCTTGGTA	ACCACTGAGGGTGCAAAAAC
**Ate1**	6940722	CAAAGTGATTCTACTGTGGCTGA	ACGAAAATCTCCAATGCAGTC
**Cul7**	3360114	CGGGACTATGCGGTGATACT	GTGGGTTCGTCTGTGGTCTT
**Psme3**	2810537	GCGAAGGTCAAACCCATAGA	GAAAGTGATGCATCCCAGGT
**Rbx1**	2340047	TTGAGGCCAGCCTACAGAGT	AGGAAAACTCCCCTGAAGGA
**Skp1a**	2450102	TGCAGCTGGGCTCTCTTAAT	GTTTCTCCACCTGGGAACAA
**Uchl1**	1230066	CCTGTCCCTTCAGTTCCTCA	GATTAACCCCGAGATGCTGA
**Huwe1**	106840041	GCTGCATTGAGACTTGAAACC	TCCACAACACAGATGCCAAT
**Tbl1x**	6400524	ATTTTCCCCCTCCCCTAATC	GAGCCTGTTCTGGATGGAAA
**Ube4b**	3610154	GCTGGAGTGGATCAGGACTC	TGGTAAGGTCAAACCCCAAA
**Ube2o**	2190040	CGGTGAGCACATTACAGCTC	GCATCATGCTTTGGCTTTTT
**Usp47**	100940601	GAATGCTTGTAAAGTCCCGTTT	CTAGCACGCTCTGCAATGAA
**Ppp2cb**	5570593	ACTGCTACCGTTGTGGGAAC	AGGTCCTGGGGAGGAATTTA
**Ube3b**	6380458	GCCTGCACAGGTAACACAGA	ACCAGGAGCTGCTGAGATGT
**Fbxo32**	110037	GGGAGGCAATGTCTGTGTTT	AAGAGGTGCAGGGACTGAGA
**Trim63**	1740164	CGACCGAGTGCAGACGATCATCTC	GTGTCAAACTTCTGACTCAGC
**Ubr5**	1780605	GCTGCCTTTGTGGAAAGTGT	TTGCAGCCAACCACAAATAA
**Asb11**	2060487	TTGTGCTGAACAAGCTCCTG	GAGGGTCCTGAATCATCCAA
**mHPRT**	-	CGTCGTGATTAGCGATGATG	TTTTCCAAATCCTCGGCATA

### Cell culture and transfection

C2C12 cells were used for transient transfection experiments. C2C12 cells were cultured in DMEM containing 20% fetal calf serum. Prior to transfection, cells were seeded on glass. Transfection was carried out in 80% cell confluence with Lipofectamine™ 2000 (Invitrogen, Breda, The Netherlands) according to the manufacturer's protocol. Plasmids used for transfection are YFP-Ala16-PABPN1 and Histone4-CFP. For the proteasome modification treatments, cells were treated 16 hours after transfection with DMSO (1:1000), 5 μM MG132 (Sigma-Aldrich St. Louis, MO, UnitedStates), or 5 U/ml IFNγ (HyCult Biotech Uden, The Netherlands) for 20 hours.

### Protein detection and Imaging

For immunocytochemistry, 16 hours post-transfection with YFP-Ala16-PABPN1, C2C12 cells were incubated with fusion medium (DMEM supplemented with 2.5% horse serum) for two days, and immunocytochemistry was performed after a short fixation [41] followed by a 15-minute incubation with 1% Triton X100, during which PABPN1 aggregates remain intact. Following antibody incubations, preparations were mounted in Citifluor (Agar Scientific Essex, United Kingdom) containing 400 μg/ml of DAPI (Sigma-Aldrich). Immunofluorescent specimens were examined with a fluorescence microscope (Leica DM RXA Vienna, Austria), 63× and 100× lens NA 1.4 plan Apo objective. Integrated intensity was measured with ImageJ http://rsbweb.nih.gov/ij/, and intensity values were corrected for background.

Antibodies used in this study are: goat anti-Asb-11 (K16) (1:1,000) Santa Cruz Biotechnology Santa Cruz, CA, United States; rabbit anti-atrogin-1 (1:1,000) ECM Biosciences Versailles, KY, United States; rabbit anti-Murf1 (1:1000) ECM Biosciences; goat anti-DDB1 (1:1000) Abcam Cambridge, United Kingdom; mouse anti-Flag (1:2000) Sigma-Aldrich; rabbit anti-Desmin MP Biomedicals (Solon, OH, United States). Alexa-Fluor 594 conjugated secondary (Invitrogen) or IRDye 680LT and 800CW conjugated secondaries (Licor Biosciences St Lincoln, NE, United States) were used for detection of first antibody.

## Abbreviations

Ala: alanine; GO: gene ontology; GT: global test.

## Competing interests

The authors declare that they have no competing interests.

## Authors' contributions

SYA and AV performed the bioinformatics studies. AV and VR performed the molecular genetics studies. Biological samples were provided by BS, BE, MS, JV, CT, GD, AC and MS. The manuscript was drafted by SYA and VR and written by SYA, PAC, SM and VR. PAC participated in the bioinformatics design, coordination and data analysis. All authors read and approved the manuscript.

## Supplementary Material

Additional file 1 **Supplementary figures and tables**. Table S1: Overview of genome-wide transcriptome microarray datasets of *Drosophila *and mouse OPMD models and muscle biopsies of OPMD patients. Table S2: Overview of muscle biopsies of OPMD patients and controls. Table S3: Literature-aided analysis of the association of biomedical concepts with OPMD-deregulated genes. Table S4: Spreading of OPMD-deregulation in UPS over the functional components and sub-classes of E3-ligases. Table S5: Direction of OPMD-deregulation over the functional components of UPS that are significantly deregulated in all organisms. Figure S1: Integrated cross-species high-throughput transcriptome study. Figure S2: Validation of expression level of selected genes from the pool of UPS OPMD-deregulated genes on the skeletal muscle of six-week-old OPMD mice, normalized to WT. Figure S3: Temporal changes in UPS gene expression. Click here for file

Additional file 2 **A list of deregulated UPS genes in mouse and human datasets**. This additional file contains t-statistics (fold change and *P-*value) for genes within the ubiquitin-proteasome pathway, in human and mouse datasets. The t-statistics represent the association of the gene expression profiles to OPMD. Click here for file

## References

[B1] Fan X Rouleau GA Progress in understanding the pathogenesis of oculopharyngeal muscular dystrophy Can J Neurol Sci 2003 30 8 14 12619777 10.1017/s0317167100002365

[B2] Blumen SC Bouchard JP Brais B Carasso RL Paleacu D Drory VE Chantal S Blumen N Braverman I Cognitive impairment and reduced life span of oculopharyngeal muscular dystrophy homozygotes Neurology 2009 73 596 601 10.1212/WNL.0b013e3181b388a3 19704078

[B3] Brais B Xie YG Sanson M Morgan K Weissenbach J Korczyn AD Blumen SC Fardeau M Tomé FM Bouchard JP The oculopharyngeal muscular dystrophy locus maps to the region of the cardiac alpha and beta myosin heavy chain genes on chromosome 14q11.2-q13 Hum Mol Genet 1995 4 429 434 10.1093/hmg/4.3.429 7795598

[B4] Brais B Bouchard JP Xie YG Rochefort DL Chretien N Tome FM Lafrenière RG Rommens JM Uyama E Nohira O Blumen S Korczyn AD Heutink P Mathieu J Duranceau A Codère F Fardeau M Rouleau GA Short GCG expansions in the PABP2 gene cause oculopharyngeal muscular dystrophy Nat Genet 1998 18 164 167 10.1038/ng0298-164 9462747

[B5] Tome FM Fardeau M Nuclear inclusions in oculopharyngeal dystrophy Acta Neuropathol 1980 49 85 87 10.1007/BF00692226 6243839

[B6] Chartier A Benoit B Simonelig M A *Drosophila *model of oculopharyngeal muscular dystrophy reveals intrinsic toxicity of PABPN1 EMBO J 2006 25 2253 2262 10.1038/sj.emboj.7601117 16642034 PMC1462976

[B7] Davies JE Wang L Garcia-Oroz L Cook LJ Vacher C O'Donovan DG Rubinsztein DC Doxycycline attenuates and delays toxicity of the oculopharyngeal muscular dystrophy mutation in transgenic mice Nat Med 2005 11 672 677 10.1038/nm1242 15864313

[B8] Catoire H Pasco MY Abu-Baker A Holbert S Tourette C Brais B Rouleau GA Parker JA Néri C Sirtuin inhibition protects from the polyalanine muscular dystrophy protein PABPN1 Hum Mol Genet 2008 17 2108 2117 10.1093/hmg/ddn109 18397876

[B9] Davies JE Sarkar S Rubinsztein DC Trehalose reduces aggregate formation and delays pathology in a transgenic mouse model of oculopharyngeal muscular dystrophy Hum Mol Genet 2006 15 23 31 10.1093/hmg/ddi422 16311254

[B10] Chartier A Raz V Sterrenburg E Verrips CT van der Maarel SM Simonelig M Prevention of oculopharyngeal muscular dystrophy by muscular expression of Llama single-chain intrabodies *in vivo* Hum Mol Genet 2009 18 1849 1859 10.1093/hmg/ddp101 19258344

[B11] Trollet C Anvar SY Venema A Hargreaves IP Foster K Vignaud A Ferry A Negroni E Hourde C Baraibar MA 't Hoen PA Davies JE Rubinsztein DC Heales SJ Mouly V van der Maarel SM Butler-Browne G Raz V Dickson G Molecular and phenotypic characterization of a mouse model of oculopharyngeal muscular dystrophy reveals severe muscular atrophy restricted to fast glycolytic fibres Hum Mol Genet 2010 19 2191 2207 10.1093/hmg/ddq098 20207626

[B12] Hino H Araki K Uyama E Takeya M Araki M Yoshinobu K Miike K Kawazoe Y Maeda Y Uchino M Yamamura K Myopathy phenotype in transgenic mice expressing mutated PABPN1 as a model of oculopharyngeal muscular dystrophy Hum Mol Genet 2004 13 181 190 10.1093/hmg/ddh017 14645203

[B13] Ioannidis JP Allison DB Ball CA Coulibaly I Cui X Culhane AC Falchi M Furlanello C Game L Jurman G Mangion J Mehta T Nitzberg M Page GP Petretto E van Noort V Repeatability of published microarray gene expression analyses Nat Genet 2009 41 149 155 10.1038/ng.295 19174838

[B14] Smyth GK Linear models and empirical Bayes methods for assessing differential expression in microarray experiments Stat Appl Genet Mol Biol 2004 3 Article3 16646809 10.2202/1544-6115.1027

[B15] Jelier R Schuemie MJ Veldhoven A Dorssers LC Jenster G Kors JA Anni 2.0: a multipurpose text-mining tool for the life sciences Genome Biol 2008 9 R96 10.1186/gb-2008-9-6-r96 18549479 PMC2481428

[B16] Goeman JJ van de Geer SA de Kort F van Houwelingen HC A global test for groups of genes: testing association with a clinical outcome Bioinformatics 2004 20 93 99 10.1093/bioinformatics/btg382 14693814

[B17] Dennis G Jr Sherman BT Hosack DA Yang J Gao W Lane HC Lempicki RA DAVID: Database for Annotation, Visualization, and Integrated Discovery Genome Biol 2003 4 3 10.1186/gb-2003-4-5-p3 PMC193660 12734009

[B18] Huang da W Sherman BT Lempicki RA Systematic and integrative analysis of large gene lists using DAVID bioinformatics resources Nat Protoc 2009 4 44 57 10.1038/nprot.2008.211 19131956

[B19] Cao PR Kim HJ Lecker SH Ubiquitin-protein ligases in muscle wasting Int J Biochem Cell Biol 2005 37 2088 2097 10.1016/j.biocel.2004.11.010 16125112

[B20] Bodine SC Latres E Baumhueter S Lai VK Nunez L Clarke BA Poueymirou WT Panaro FJ Na E Dharmarajan K Pan ZQ Valenzuela DM DeChiara TM Stitt TN Yancopoulos GD Glass DJ Identification of ubiquitin ligases required for skeletal muscle atrophy Science 2001 294 1704 1708 10.1126/science.1065874 11679633

[B21] Sandri M Signaling in muscle atrophy and hypertrophy Physiology (Bethesda) 2008 23 160 170 18556469 10.1152/physiol.00041.2007

[B22] Reyes-Turcu FE Ventii KH Wilkinson KD Regulation and cellular roles of ubiquitin-specific deubiquitinating enzymes Annu Rev Biochem 2009 78 363 397 10.1146/annurev.biochem.78.082307.091526 19489724 PMC2734102

[B23] Finley D Recognition and processing of ubiquitin-protein conjugates by the proteasome Annu Rev Biochem 2009 78 477 513 10.1146/annurev.biochem.78.081507.101607 19489727 PMC3431160

[B24] Abu-Baker A Messaed C Laganiere J Gaspar C Brais B Rouleau GA Involvement of the ubiquitin-proteasome pathway and molecular chaperones in oculopharyngeal muscular dystrophy Hum Mol Genet 2003 12 2609 2623 10.1093/hmg/ddg293 12944420

[B25] Victor M Hayes R Adams RD Oculopharyngeal muscular dystrophy. A familial disease of late life characterized by dysphagia and progressive ptosis of the evelids N Engl J Med 1962 267 1267 1272 10.1056/NEJM196212202672501 13997067

[B26] Calado A Tomé FM Brais B Rouleau GA Kuhn U Wahle E Carmo-Fonseca M Nuclear inclusions in oculopharyngeal muscular dystrophy consist of poly(A) binding protein 2 aggregates which sequester poly(A) RNA Hum Mol Genet 2000 9 2321 2328 11001936 10.1093/oxfordjournals.hmg.a018924

[B27] Tavanez JP Calado P Braga J Lafarga M Carmo-Fonseca M *In vivo *aggregation properties of the nuclear poly(A)-binding protein PABPN1 RNA 2005 11 752 762 10.1261/rna.7217105 15811916 PMC1370760

[B28] Kloetzel PM Ossendorp F Proteasome and peptidase function in MHC-class-I-mediated antigen presentation Curr Opin Immunol 2004 16 76 81 10.1016/j.coi.2003.11.004 14734113

[B29] Ferrington DA Husom AD Thompson LV Altered proteasome structure, function, and oxidation in aged muscle FASEB J 2005 19 644 646 15677694 10.1096/fj.04-2578fje

[B30] Osna NA Clemens DL Donohue TM Jr Interferon gamma enhances proteasome activity in recombinant Hep G2 cells that express cytochrome P4502E1: modulation by ethanol Biochem Pharmacol 2003 66 697 710 10.1016/S0006-2952(03)00252-1 12948850

[B31] Hoeller D Dikic I Targeting the ubiquitin system in cancer therapy Nature 2009 458 438 444 10.1038/nature07960 19325623

[B32] Liu Z Miers WR Wei L Barrett EJ The ubiquitin-proteasome proteolytic pathway in heart vs skeletal muscle: effects of acute diabetes Biochem Biophys Res Commun 2000 276 1255 1260 10.1006/bbrc.2000.3609 11027619

[B33] Combaret L Dardevet D Bechet D Taillandier D Mosoni L Attaix D Skeletal muscle proteolysis in aging Curr Opin Clin Nutr Metab Care 2009 12 37 41 10.1097/MCO.0b013e32831b9c31 19057185

[B34] Taillandier D Combaret L Pouch MN Samuels SE Bechet D Attaix D The role of ubiquitin-proteasome-dependent proteolysis in the remodelling of skeletal muscle Proc Nutr Soc 2004 63 357 361 10.1079/PAR2004358 15294055

[B35] Ciechanover A Brundin P The ubiquitin proteasome system in neurodegenerative diseases: sometimes the chicken, sometimes the egg Neuron 2003 40 427 446 10.1016/S0896-6273(03)00606-8 14556719

[B36] Vignaud A Ferry A Huguet A Baraibar M Trollet C Hyzewicz J Butler-Browne G Puymirat J Gourdon G Furling D Progressive skeletal muscle weakness in transgenic mice expressing CTG expansions is associated with the activation of the ubiquitin-proteasome pathway Neuromuscul Disord 2010 20 319 325 10.1016/j.nmd.2010.03.006 20346670

[B37] Lee CK Klopp RG Weindruch R Prolla TA Gene expression profile of aging and its retardation by caloric restriction Science 1999 285 1390 1393 10.1126/science.285.5432.1390 10464095

[B38] Corbeil-Girard LP Klein AF Sasseville AM Lavoie H Dicaire MJ Saint-Denis A Pagé M Duranceau A Codère F Bouchard J-P Karpati G Rouleau GA Massie B Langelier Y Brais B PABPN1 overexpression leads to upregulation of genes encoding nuclear proteins that are sequestered in oculopharyngeal muscular dystrophy nuclear inclusions Neurobiol Dis 2005 18 551 567 10.1016/j.nbd.2004.10.019 15755682

[B39] Smyth GK Speed T Normalization of cDNA microarray data Methods 2003 31 265 273 10.1016/S1046-2023(03)00155-5 14597310

[B40] Li W Bengtson MH Ulbrich A Matsuda A Reddy VA Orth A Chanda SK Batalov S Joazeiro CA Genome-wide and functional annotation of human E3 ubiquitin ligases identifies MULAN, a mitochondrial E3 that regulates the organelle's dynamics and signaling PLoS One 2008 3 e1487 10.1371/journal.pone.0001487 18213395 PMC2198940

[B41] Raz V Carlotti F Vermolen BJ van der Poel E Sloos WC Knaan-Shanzer S de Vries AA Hoeben RC Young IT Tanke HJ Garini Y Dirks RW Changes in lamina structure are followed by spatial reorganization of heterochromatic regions in caspase-8-activated human mesenchymal stem cells J Cell Sci 2006 119 4247 4256 10.1242/jcs.03180 17003109

