# Supplementary material for: Deregulation of the ubiquitin-proteasome system is the predominant molecular pathology in OPMD animal models and patients
Source: Skelet Muscle. 2011 Apr 4;1:15. doi: 10.1186/2044-5040-1-15 (PMC3156638; doi:10.1186/2044-5040-1-15)

<u>SUPPLEMENTARY MATERIALS</u>

Deregulation of the ubiquitin-proteasome system is the predominant molecular pathology in OPMD animal models and patients

SY Anvar[1], PAC ‘t Hoen[1], A Venema[1], B van der Sluijs[2], B van Engelen[2], M Snoeck[3], J Vissing[4], C Trollet[5,6], G Dickson[5], A Chartier[7], M Simonelig[7], GJB van Ommen[1], SM van der Maarel[1], V Raz[1,*]

[1]Center for Human and Clinical Genetics, Leiden University Medical Center, P.O. Box 9600, 2300 RC Leiden, the Netherlands. [2]Radboud University Nijmegen Medical Centre, Department of Neurology, Nijmegen, the Netherlands. [3]Department of Anaesthesia, Canisius-Wilhelmina Hospital, Nijmegen, the Netherlands. [4]Neuromuscular Research Unit and Department of Neurology, University of Copenhagen, Rigshospitalet, Denmark. [5]School of Biological Sciences, Royal Holloway – University of London, Surrey, TW20 0EX, United Kingdom. [6]INSERM U974, UMR 7215 CNRS, Institut de Myologie, UM 76 Université Pierre et Marie Curie, Paris, France. [7]Institut de Genetique Humaine, CNRS UPR1142, 141 rue de la Cardonille, 34396 Montpellier Cedex 5, France

Supplementary Table 1: Overview of genome-wide transcriptome microarray datasets of *Drosophila* and mouse OPMD models and muscle biopsies of OPMD patients.

Biological Systems	Condition and Control	Tissue	Number of Samples		Age	MA Platform
**Drosophila**	bovine PABPN1 overexpression in muscle vs. wild-type	Adult thoracic muscles	3	pools of 50 flies per genotype	1 day<br>6 days<br>11 days	15K INDAC spotted oligonucleotide array
**Mouse**	hPABPN1 overexpression in muscle A17.1 mice vs. wild-type	Quadriceps	6	replicates per genotype	6 weeks<br>18 weeks<br>26 weeks	Illumina 48K Mouse v.1 bead array
**Human**	OPMD patients vs. control subjects	Quadriceps	9<br>13<br>39	Pre-symptomatic<br>Symptomatic<br>Controls	17 – 22 years control<br>31 – 40 years Pre-Symptomatic<br>38 – 42 years control<br>49 – 60 years symptomatic<br>58 – 67 years control	Illumina 48K Human v.3 bead array

Supplementary Table 2: Overview of muscle biopsies of OPMD patients and controls. All patients are heterozygous expPABPN1 carriers as indicated by sequence analysis.

	Sex	Age	GCG Mutation	Muscle Histology
Pre-Symptomatic	Female	39	12/6	Sporadic atrophic fibre
	Female	37	10/6	Moderate dystrophic alterations
	Female	37	12/6	Normal
	Female	31	9/6	Slight dystrophic alteration
Symptomatic	Female	60	9/6	Moderate dystrophic alterations
	Female	49	10/6	Moderate dystrophic alterations
	Male	59	10/6	Moderate dystrophic alterations
	Female	57	11/6	Severe dystrophic alterations

Supplementary Figure 1: Integrated cross-species high-throughput transcriptome study.

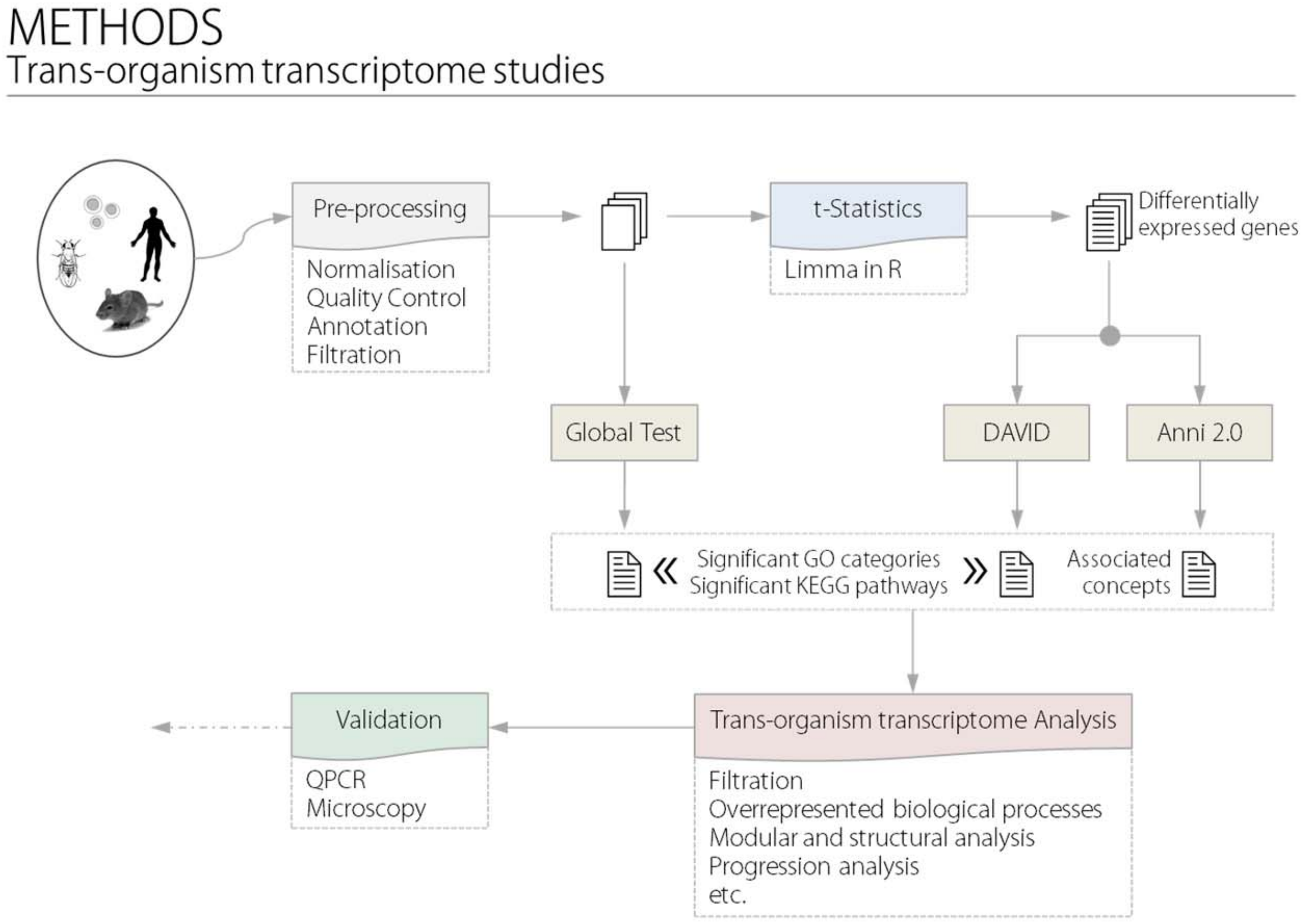

Supplementary Table 3: Literature-aided analysis of the association of biomedical concepts with OPMD-deregulated genes.

	Drosophila		Mouse		Human
1	ribosomal protein activity	1	*Ubiquitination*	1	*ubiquitin activity*
2	*ubiquitin activity*	2	*Ubiquitins*	2	RNA Binding
3	RNA Binding	3	*Ubiquitin*	3	*Ubiquitination*
4	*Ubiquitination*	4	*Ligase*	4	RNA Splicing
5	polyethylene glycol monostearate	5	SNAP receptor	5	GTP Binding
6	*Ligase*	6	Internal Ribosome Entry Site	6	protein transport
7	POLR2F	7	Phosphotransferases	7	Alternative Splicing
8	GTP Binding	8	*Cullin Proteins*	8	Transcription, Genetic
9	ribosome biogenesis and assembly	9	Muscle Proteins	9	intracellular protein transport
10	Ribosome Subunits	10	Mitogen-Activated Protein Kinases	10	GTP-binding

Supplementary Figure 2: Validation of expression level of selected genes from the pool of UPS OPMD-deregulated genes on the skeletal muscle of 6 weeks-old OPMD mice, normalized to WT. Histograms indicate the log2(ratio) of the measured expression values using RT Q-PCR (grey bars) and microarray (black bars) for 4 WT and 6 OPMC mice (* *P* <0.05).

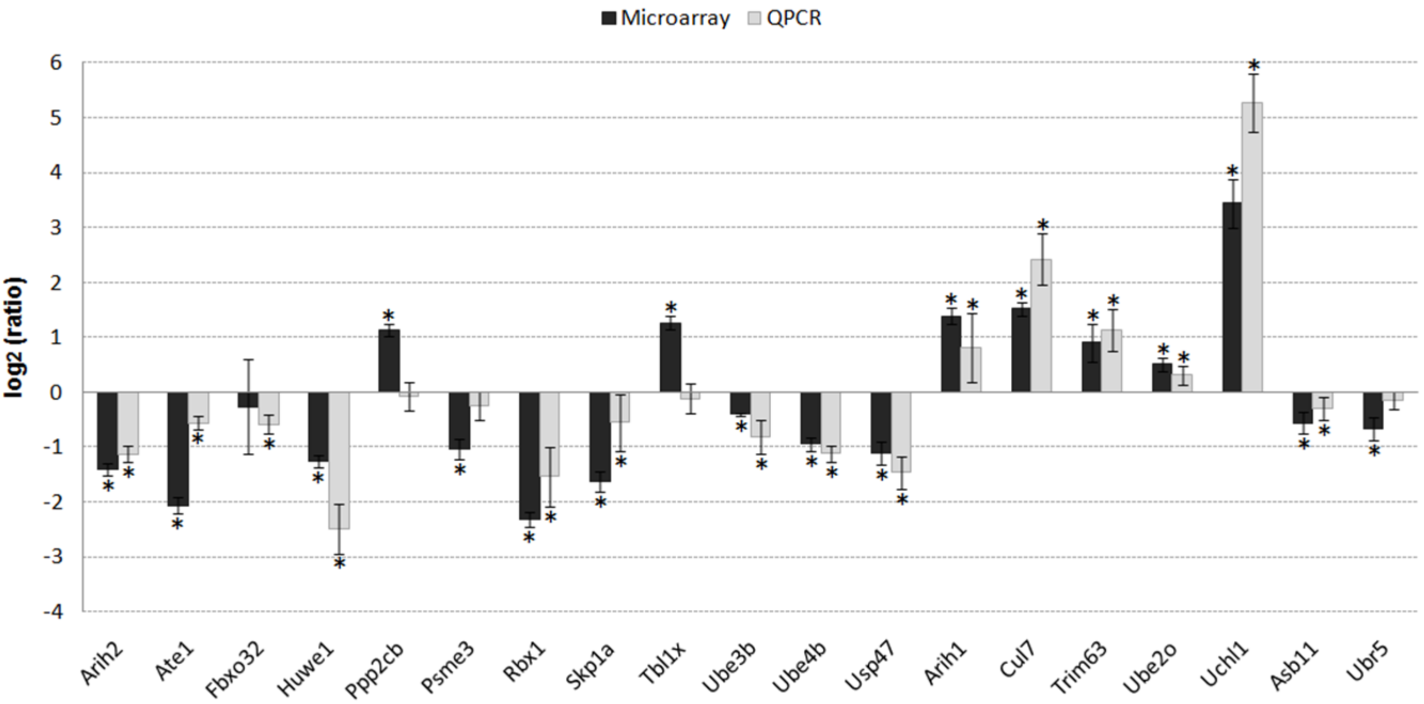

Supplementary Table 4: Spreading of OPMD-deregulation in UPS over the functional components and sub-classes of E3-ligases. The number of total genes in each component is shown; the percentage of OPMD-deregulated (D.E.) genes and *P*-values are indicated. For *Drosophila* and mouse, *P*-values indicate the significance of OPMD-deregulation in combined datasets from three time-points and the percentage of D.E. genes represents an average across three time-points. The overlap in OPMD-deregulated genes between human and mouse is shown and the percentage of deregulated genes shows the fraction of overlap in human D.E. genes.

	**Drosophila**			**Mouse**			**Human**			**Overlap** *Mouse vs. Human*	
	# Total Genes	% D.E. Genes	*P*-Value (FDR)	# Total Genes	% D.E. Genes	*P*-Value (FDR)	# Total Genes	% D.E. Genes	*P*-Value (FDR)	# D.E. Genes	% D.E. Genes
Ubiquitin	2	50.00	4.18E-05	3	11.11	1.28E-01	3	33.33	1.19E-01	0	00.00
E1 Ubiquitin Activation	4	16.67	1.24E-01	7	04.76	7.29E-02	7	14.29	7.94E-02	0	00.00
E2 Ubiquitin Conjugation	22	13.64	9.19E-06	33	44.42	1.51E-08	34	23.53	7.31E-02	3	37.50
E3 Ubiquitin Ligase	249	13.99	1.92E-04	526	29.58	1.64E-08	586	24.74	4.35E-03	69	47.59
RING	226	13.61	1.29E-04	495	28.13	1.67E-08	550	23.04	7.83E-03	60	47.35
HECT	16	22.92	2.49E-02	23	42.00	1.68E-08	28	32.14	1.59E-02	7	77.78
U-Box	7	4.76	6.98E-01	8	46.00	2.52E-08	8	37.50	7.94E-03	2	66.67
Deubiquitination (DUB)	35	20.00	1.63E-05	72	45.83	1.48E-08	75	24.00	3.15E-02	13	72.22
Proteasome	47	29.79	2.15E-04	37	36.94	1.37E-07	37	51.35	9.27E-03	11	57.90

Supplementary Table 5: Direction of OPMD-deregulation over the functional components of UPS that are significantly deregulated in all organisms.

	Mouse				Human			
	# Total Genes	% D.E. Genes	↑	↓	# Total Genes	% D.E. Genes	↑	↓
E2 Ubiquitin Conjugation	33	44.42	50.00	50.00	34	23.53	50.00	50.00
E3 Ubiquitin Ligase	526	29.58	51.44	48.56	586	24.74	50.00	50.00
Deubiquitination (DUB)	72	45.83	42.00	58.00	75	24.00	41.18	58.82
Proteasome	37	36.94	59.38	40.62	37	51.35	42.86	57.14

Supplementary Table 6: Deregulation of autophagy and lysosome as compared to proteasome in OPMD model systems and OPMD patients.

	Drosophila			Mouse			Human			Overlap *Mouse vs. Human*	
	# Total Genes	% D.E. Genes	*P*-Value (FDR)	# Total Genes	% D.E. Genes	*P*-Value (FDR)	# Total Genes	% D.E. Genes	*P*-Value (FDR)	# D.E. Genes	% D.E. Genes
Proteasome	47	29.79	2.15E-04	37	36.94	1.37E-07	37	51.35	9.27E-03	11	57.90
Autophagy	16	25.00	1.07E-03	39	30.77	8.13E-08	32	18.75	1.37E-02	1	16.67
Lysosome	60	5.00	1.64E-02	75	25.33	6.06E-03	73	24.66	1.54E-02	6	33.33

Supplementary Figure 3: Temporal changes in UPS gene expression. A linear regression was applied to identify temporal changes in expression levels of OPMD-deregulated genes in mouse. **A)** Histogram shows the percentage of age associated OPMD-deregulated genes for each of the UPS functional components and E3-ligase subclasses. **B)** OPMD-deregulated genes showing age and OPMD associated progression. Histogram shows the percentage of genes with age and OPMD associated expression for each of the functional components. The number of genes in each bar is indicated.

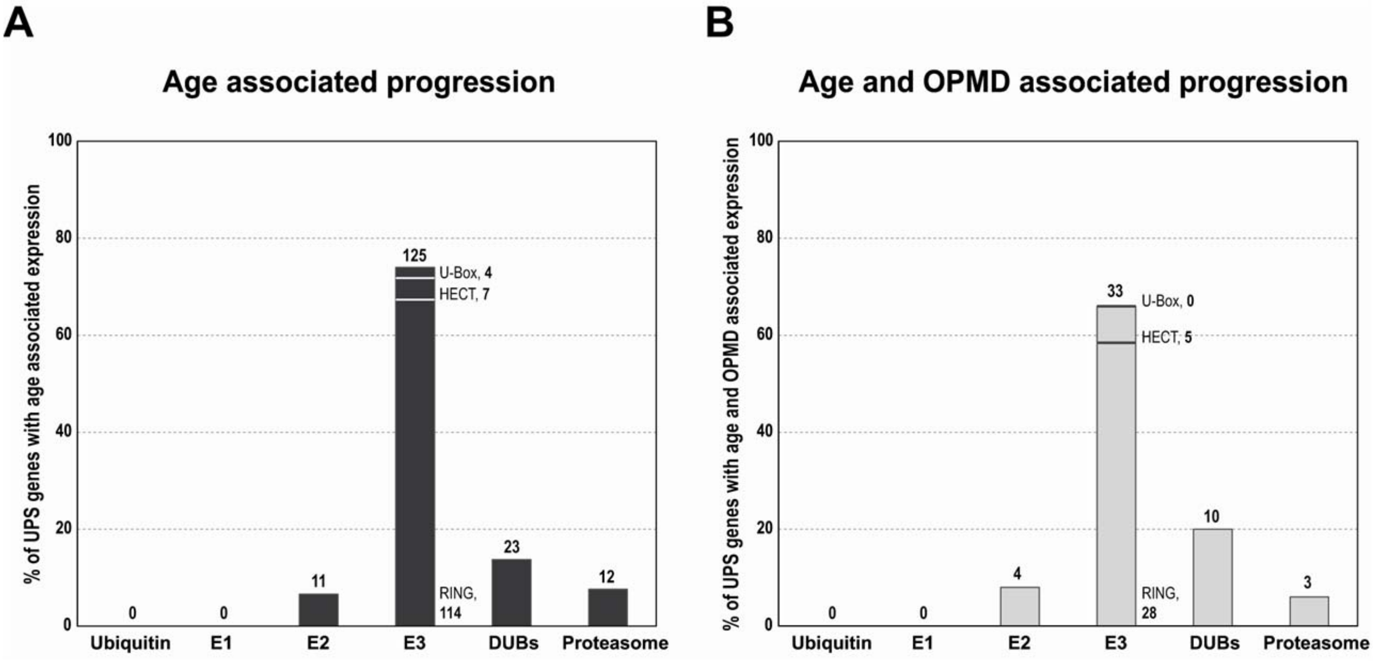

Supplement: Additional file 1 — Supplementary figures and tables. Table S1: Overview of genome-wide transcriptome microarray datasets of Drosophila and mouse OPMD models and muscle biopsies of OPMD patients. Table S2: Overview of muscle biopsies of OPMD patients and controls. Table S3: Literature-aided analysis of the association of biomedical concepts with OPMD-deregulated genes. Table S4: Spreading of OPMD-deregulation in UPS over the functional components and sub-classes of E3-ligases. Table S5: Direction of OPMD-deregulation over the functional components of UPS that are significantly deregulated in all organisms. Figure S1: Integrated cross-species high-throughput transcriptome study. Figure S2: Validation of expression level of selected genes from the pool of UPS OPMD-deregulated genes on the skeletal muscle of six-week-old OPMD mice, normalized to WT. Figure S3: Temporal changes in UPS gene expression. [file 2044-5040-1-15-S1.PDF]
